# Effects of Fiber and Surface Treatment on Airport Pavement Concrete against Freeze–Thawing and Salt Freezing

**DOI:** 10.3390/ma15217528

**Published:** 2022-10-27

**Authors:** Lei Xu, Yong Lai, Daoxun Ma, Junjie Wang, Molan Li, Le Li, Zhibin Gao, Yan Liu, Pukang He, Yi Zhang

**Affiliations:** 1 Department of Civil Engineering, Tsinghua University, Beijing 100084, China; 2 Beijing Super-Creative Technology Co., Ltd., Beijing 100621, China; 3 China Airport Construction Group Corporation of CACC, Beijing 100621, China; 4 Capital Airport Group Co., Ltd., Beijing 100621, China

**Keywords:** fiber-reinforced concrete, freeze–thawing, salt freezing, airport pavement, silane, spraying and immersion

## Abstract

Airport pavement concrete often suffers from freeze–thawing damage in high latitude and cold areas. In addition, the use of aircraft deicer makes the airport pavement concrete suffer from salt-freezing damage. To improve the durability of airport pavement concrete, modified polyester synthetic fiber (FC), cellulose fiber (CF), and basalt fiber (BF) reinforced concrete were prepared in this paper. The mechanical strength, pore structure, and frost resistance (freeze–thawing and salt freezing) of fiber-reinforced concrete were investigated. The effects of the combined action of fiber (fiber type and content) and surface treatment methods (spraying silane and impregnating silane) on the frost resistance of concrete were investigated. The results show that the flexural strength of concrete is positively correlated with the elastic modulus of fiber, but has little effect on the compressive strength. Fiber can reduce mass loss and dynamic modulus loss of concrete subjected to frost damage. FC more effectively improved the frost resistance of concrete than CF. After 30 cycles of salt freezing, the spalling amount of concrete sprayed or soaked with silane was decreased by 65.5% and 55.5%, respectively. Adding fiber and impregnating silane reduced the spalled concrete by up to 70.5%. Spraying silane treatment is better than impregnating silane treatment in enhancing the frost resistance of concrete because a better silane condensation reaction is achieved with spraying silane.

## 1. Introduction

As one of the most basic and important infrastructures of the airport, airport pavement has a significant impact on flight safety and airport operation efficiency. In addition to geometric factors such as length, width, and slope, airport pavement must also meet the requirements of strength, stiffness, flatness, and durability. Cement concrete pavement is the main structural form of airport pavement, which is affected repeatedly by various environmental climates. For example, the pavement concrete of some airports in high-latitude cold areas is damaged by freezing and thawing all year round, resulting in performance degradation, shedding, and failure [1,2,3,4,5].

In addition to the common failure form of gradual cracking, denudation, and structural failure under the action of repeated freeze–thaw cycles, airport pavement is also damaged by surface denudation caused by freezing in the presence of deicing salt (glycol aircraft deicing fluid). Some concrete with good water-resistant freeze–thaw damage tests will also suffer serious salt freeze–thaw erosion after being sprinkled with deicing salt for several winters, and the pavement aggregate will be exposed, which will seriously affect its use [6,7]. The typical damage of salt freezing gradually extends from the surface to the inside. The surface mortar is peeled off and the aggregate is exposed, but the concrete under the denudation layer is basically hard and intact, and layered denudation traces can be seen on the damaged section.

To improve the flexural strength and frost resistance of pavement materials, fibers are often added to concrete to improve the brittleness and durability of concrete and prevent damage caused by various factors [8,9,10,11]. Fiber has a significant effect on the mechanical properties of concrete under freeze–thaw conditions, and the improvement effect of different types and contents of fiber is different [12,13,14]. Many other types of composite concrete and applications are also proposed to improve concrete durability [15,16].

In addition to the durability of fiber-reinforced concrete, since the 1980s, the United States has used organosilane materials to spray and soak the concrete structures of roads and bridges to increase the durability of structures. Many durability technical standards also take surface silane impregnation as an effective surface protection measure [17,18,19]. Wang et al. and Jiang et al. systematically studied the effects of silane brushing times, concrete curing age, construction temperature, mix proportion, and other conditions on the silane impregnation effect [20,21].

In summary, at present, research on freeze–thaw damage of fiber-reinforced concrete and silane impregnation protection has been carried out, but research on the protection of concrete against freeze–thaw damage by different fiber types, dosages, and different surface treatment methods is still limited. This study attempts to fill this gap through the mechanical test, freeze–thaw test, and salt-freezing test, combined with SEM, XRD, and MIP measurements.

## 2. Experimental Program

### 2.1. Raw Materials

Cement: The cement is P.O 42.5 ordinary Portland cement (conforming to Chinese national standard GB175-2007) with a specific surface area of 335 m^2^/kg [22]. The initial and final setting times were 180 min and 235 min, the flexural and compressive strengths were 4.9 MPa and 22.5 MPa at 3 days, and the flexural and compressive strengths were 8.6 MPa and 48.3 MPa at 28 days, respectively. All these data are from the cement plant (Fushun Aosaier Technology Co., Ltd., Fushun, China).

**Figure 1:**
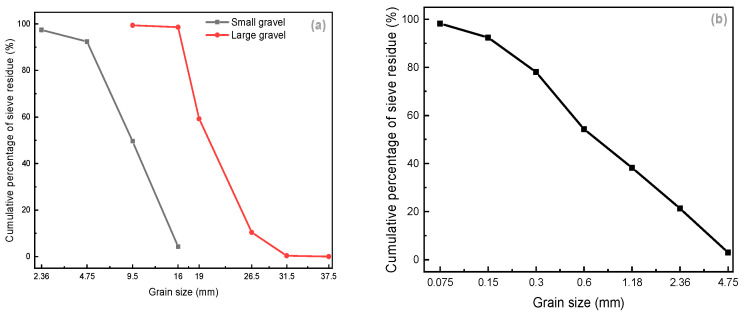
Coarse aggregate (**a**) and fine aggregate (**b**) sieving curve.

Coarse Aggregates: The coarse aggregates are subdivided into large (16–31.5 mm) and small (4.75–16 mm) gravel and both of them are well graded; the properties are shown in Table 1 and sieving curves are shown in Figure 1a.

Fine Aggregates: The fine aggregates were natural sand (0–4.75 mm), whose apparent density was 2.655 g/cm^3^, fineness modulus was 2.3, sand mud content was 2.9%, and its sieving curve is shown in Figure 1b.

**Figure 2:**
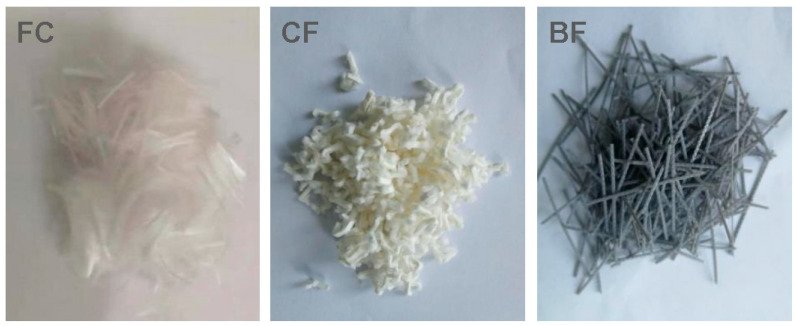
Morphology of the three types of fibers.

Fibers: Three types of fiber were used in this study (provided by Beijing Zhongqi Zhuochuang Technology Development Co., Ltd., Beijing, China). Ultra-high strength modified polyester synthetic fiber (FC) is a new material specially developed for airport road surface concrete. Cellulose fiber (CF) is a sheet-like monomer of fiber monofilament. Basalt fiber (BF) is a continuous fiber drawn from natural basalt. The morphology of the three types of fibers is shown in Figure 2, and their physical and mechanical properties are shown in Table 2. The results of the fiber characteristic test are provided by the corresponding manufacturers.

Additives and other chemical reagents: The superplasticizer is an air-entraining polycarboxylic acid water-reducing agent, which is in accordance with the provisions of GB8076-2008 Concrete Admixtures [23]. The glycol deicing liquid used for the salt-freezing test is FCY-1A-SAE Type Ⅰ aircraft deicing anti-icing liquid, which is a light yellow transparent liquid with a specific gravity of 1.1120 ± 0.015 at 20 °C. Silane for impregnation and spraying is a compound of a variety of long-chain silanes. All water used in this experiment was tap water.

### 2.2. Mix Design and Specimen Preparation

To ensure the uniformity of the concrete mixture, concrete was mixed with a forced mixer. Test samples were manufactured in the laboratory. The mixing procedure of fiber concrete used in the test is as follows: first pour stones, sand, cement, and fiber into the mixer successively, mixing for 120 s, then pour the mixed liquid of water reducer and water into the mixer, mixing for another 120 s, and cast the test block after the fiber is evenly dispersed. The test samples were filled at one time when forming, and then placed on the vibration table for 10 s. After vibration, the surface was troweled, and the samples were water cured (20 °C) after demoulding at 24 h.

The concrete mix consists of eight mix groups according to different fiber content and fiber types, whether a water-reducing agent is used or not, and different sand ratios. Their mixed proportion and relevant composition information are shown in Table 3 below. There are 8 recipes in total, labeled G1 to G8. The water to cement ratio was kept constant at 0.4. The sand ratio is the percentage of the sand mass in the total mass of sand and stone in the concrete. In this experiment, the sand ratio was controlled to be 0.3. Air entraining and water-reducing superplasticizer with a solid content of 2% (relative to the mass of cement), i.e., 6.6 kg/m^3^, was used in the concrete mixes except for G7. 

### 2.3. Mechanical Tests

After mixing, prismatic specimens of 150 × 150 × 550 mm^3^ were molded and compacted according to the GBT 50081-2019 [24]. After 24 h of being protected with a plastic film, the specimens were demoulded and placed in a wet chamber where they were water cured until 28 days. Three specimens in each mixed group were tested for flexural strength according to GBT 50081-2019 [24]. The two halves resulting from the flexural tests were then tested for compressive strength according to the same standard.

### 2.4. Freeze–Thaw Cycle Test

The freeze–thaw cycle test was carried out according to a quick freezing method in GB 50082-2009 [25], which simulated frost damage to the airport pavement in cold regions. The size of the sample was 100 × 100 × 400 mm prismatic specimen. At the age of 28 days, the specimen soaked in water was taken out and directly used for the test. After different freeze–thaw cycles, the loss of mass and dynamic modulus were measured.

### 2.5. Salt-Freezing Test

**Figure 3:**
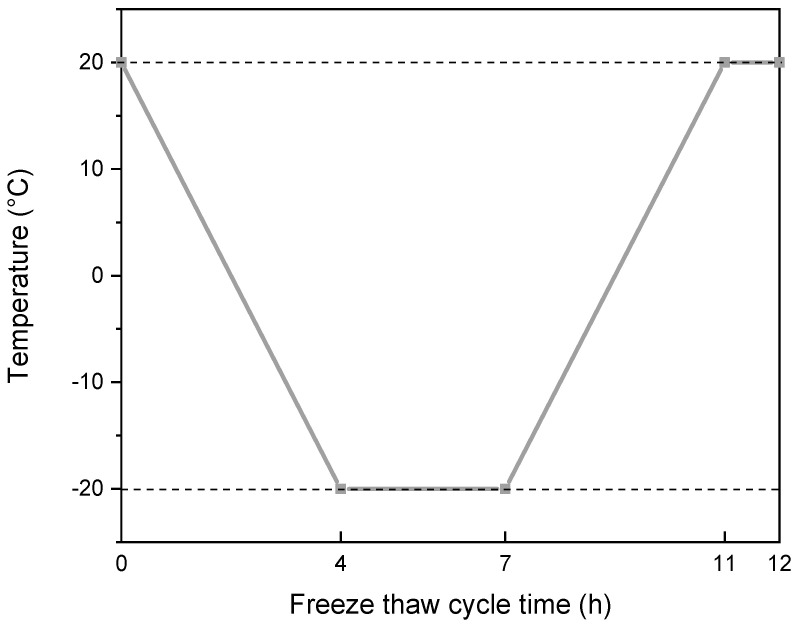
One typical cycle in a freeze–thaw cycle program.

The salt-freezing test was carried out according to MH 5006-2015 [26], and the experiment was based on the single-side salt-freezing test in the freezing–thawing damage test [27,28]. The test specimen for the salt freezing were cylindrical, and the size was Φ150 mm × 100 mm. Except for the exposure surface, epoxy resin was applied on the other surfaces of the specimen after 28 days of curing. The salt-freezing solution was prepared with 97% distilled water and 3% ethylene glycol. The working temperature was −20 °C to 20 °C. The measurement, weighing, and appearance inspection of the test piece was carried out instantly, and the piece to be tested was covered with wet cloth. The single-sided freeze–thaw test box automatically carried out the freeze–thaw cycle according to the predefined freeze–thaw cycle program. The one typical cycle in the freeze–thaw cycle program is shown in Figure 3 below.

### 2.6. Mercury Intrusion Porosimetry (MIP)

After the concrete samples were cured to the specified age, they were crushed with a small hammer. The selected pastes, particles about the size of soybeans, were soaked in sufficient absolute ethanol for 24 h to stop hydration, and then placed in a vacuum drying oven at 40 °C for vacuum drying for 3 days. After that, the pore structure was tested by the Autopore IV9510 mercury intrusion instrument (McMurray Teck Instrument Co., Ltd., Atlanta, GA, USA). A selection of 3–5 g samples were taken for each test, the pressure range was 0.0036–414 MPa, and the measured minimum pore diameter was about 3 nm.

### 2.7. X-ray Diffraction (XRD)

XRD patterns were collected by using a Bruker D8 powder diffractometer (Bruker, Germany) with a CuKα radiation source (λ = 1.5418 Å) at 40 kV and 40 mA to identify the variation in the crystalline phases. Scanning angles (2θ) were from 5° to 70° at a rate of 0.6 s/step and 0.02° per step. The samples for XRD were dried at 40 °C for 2 days and then ground to powder (≤75 μm).

### 2.8. Scanning Electron Microscopy (SEM)

Field emission environmental scanning microscope (FEI QUANTA 200 FEG, USA) and a working voltage of 15.0 kV was used for the SEM test to observe the micromorphology of the samples under high vacuum mode. The sample preparation method is as follows: use the cutting machine to cut the large test piece into small pieces, and then crush it before observations. The fresh broken surface, which was the target surface, was coated with carbon before being placed into the measurement chamber.

## 3. Results

### 3.1. Compressive and Flexural Strength of Concrete

**Figure 4:**
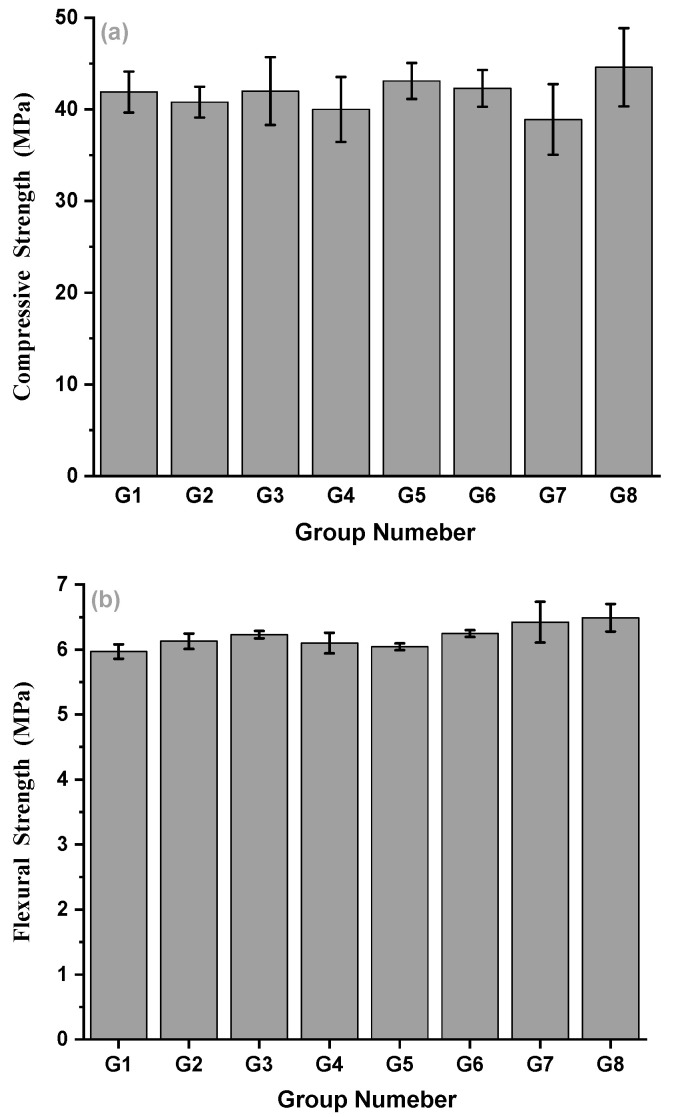
Compressive strength (**a**) and flexural strength (**b**) at 28 days.

Figure 4 shows the compressive and flexural strength results of different concrete mixes. From the results of G3 and G7, the effect of the superplasticizer on the strength can be observed. G3 and G8 groups have different sand ratios. Moreover, the effect of fiber variety was investigated (G3, G5, and G6) and can be compared with G1 without fiber. In the mixes with different FC fiber contents (0 kg/m^3^, 1.0 kg/m^3^, 1.4 kg/m^3^, and 1.8 kg/m^3^), the effect of fiber content on the strength of concrete can be discussed. The compressive strength and flexural strength of the eight groups, G1–G8, at 28 days are shown in Figure 4a,b.

From the experimental results, the addition of superplasticizer (G3) increases the 28 d compressive strength of concrete but decreases the 28 d flexural strength of concrete. Compared with G3 and G8, the sand ratio is reduced from 0.3 to 0.28, and the compressive and flexural strength of concrete at 28 days is increased by 6.19% and 8.7%, respectively. The compression failure of concrete may include the failure of the aggregate itself in addition to the bonding interface between the cement paste itself and the aggregate. Then, a larger sand ratio could reduce the amount of coarse aggregate and the ability of aggregate in concrete to bear its damage, resulting in a reduction in compressive strength.

When the fiber type changes but the fiber content is relatively low, the 28 d compressive strength of fiber concrete changes little, and the effect of improving the compressive strength of the fiber concrete is not obvious. Among the three fibers, CF (G5) showed the highest compressive strength. Compared with G1 (without fiber), the flexural strength of G3 (FC), G5 (CF), and G6 (BF) at 28 days increased by 4.36%, 1.17%, and 4.64%, respectively. After adding fiber, the 28 d flexural strength of the concrete is improved. The flexural strength of the high modulus fiber (FC) concrete G3 was the highest, the flexural strength of the medium modulus fiber (BF) concrete G6 was the middle one, and the flexural strength of the low modulus fiber (CF) concrete G5 was the lowest. The flexural strength of fiber-reinforced concrete was positively correlated with the elastic modulus of fiber. The 28 d flexural strength of all specimens was greater than 5.75 MPa, which meets the requirements of flexural strength of airport pavement concrete [23].

The change of FC content from G1–G4 is 0 kg/m^3^, 1.0 kg/m^3^, 1.4 kg/m^3^, and 1.8 kg/m^3^, respectively. From the experimental results, the 28 d compressive strength of concrete does not change significantly and fluctuates within a reasonable range, among which the 28 d compressive strength of G3 is greater. The flexural strength of the three groups with 1.0 kg/m^3^, 1.4 kg/m^3^, and 1.8 kg/m^3^ fibers increased by 2.65%, 4.36%, and 2.18%, respectively.

### 3.2. Frost Resistance of Fiber Reinforced Concrete under Freeze–Thawing

**Figure 5:**
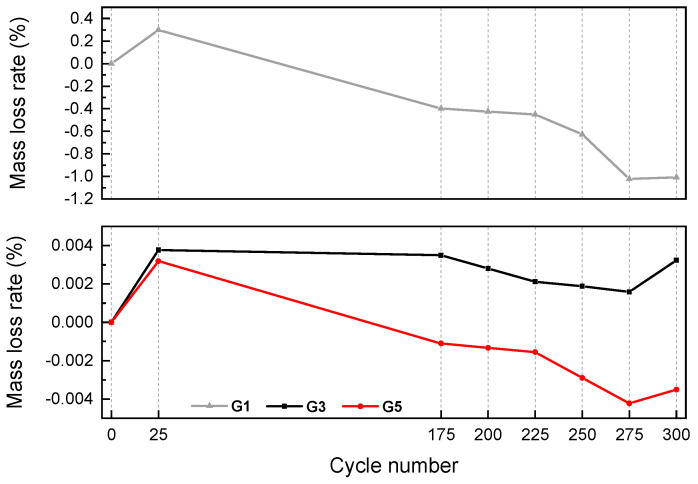
Mass loss after different freeze–thaw cycles (note: the plus and minus directions of the Y axis represent the increase and decrease in mass, respectively).

**Figure 6:**
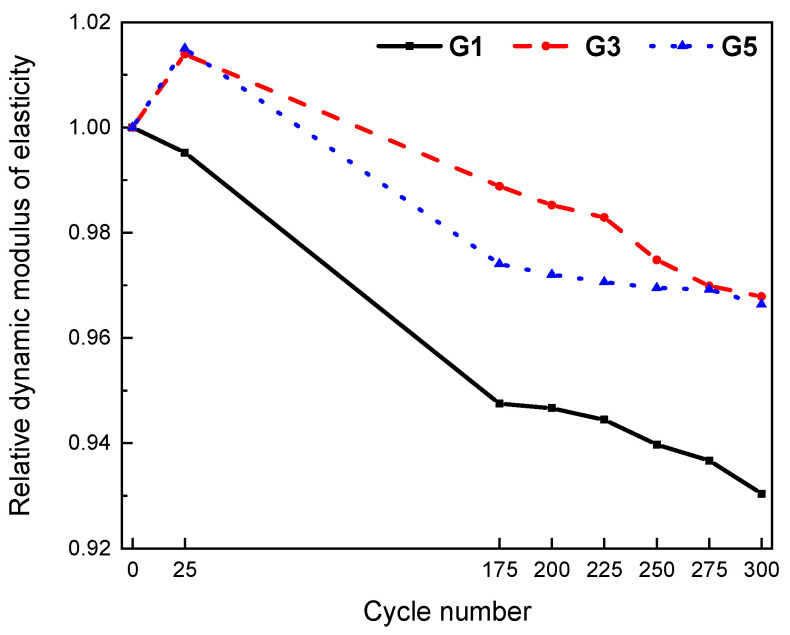
Relative dynamic elastic modulus loss after different freeze–thaw cycles.

From the above mechanical results, three groups G1, G3, and G5 were selected for freeze–thaw cycle experiments. The mass loss and relative dynamic elastic modulus under different freeze–thaw cycles are shown in Figure 5 and Figure 6, respectively.

After 200 freeze–thaw cycles, the surface of the reference concrete (G1) specimen showed local peeling and dropping. The spalling phenomenon on the surface of fiber-reinforced concrete was not obvious. Compared with the FC concrete, the surface holes of the CF concrete were more obvious. After 300 freeze–thaw cycles, the surface of the reference concrete (G1) specimen showed flake falling, the number of new holes increased, and the denudation was obvious. Individual new holes appeared in the FC concrete. The original holes on the surface of the CF-fiber-reinforced concrete specimens increased, but there was still less spalling than the reference concrete specimens. The different trend from cycles 275 to 300 could be explained by the fact that after 275 freeze–thaw cycles, the mass peels off completely. Therefore, 275 to 300 cycles may cause the freeze–thaw water absorption icing mass to be greater than the peeling mass, resulting in a mass increase.

In G1 without fiber, the mass loss of 300 freeze–thaw cycles was about 1%, and there was almost no mass loss in G3 and G5. It is obvious that the use of fiber can improve the frost resistance of concrete. Among them, compared with CF, FC is more effective in improving the spalling mass loss of concrete caused by frost resistance.

Similar to the experimental results of mass loss rate, the relative dynamic elastic modulus of the two groups with fibers was greater than that of the control group without fiber. After 300 freeze–thaw cycles, the relative dynamic elastic modulus of the control group (G1) was reduced to 0.93, while the relative dynamic elastic modulus of G3 and G5 was reduced to 0.968 and 0.966, respectively. The improvement increment of the relative dynamic elastic modulus was 4.03% and 3.86%, respectively. Compared with the concrete with CF, the frost resistance of concrete with FC is better.

### 3.3. Effect of Surface Treatment Methods on the Salt Freezing of Fiber-Reinforced Concrete

**Figure 7:**
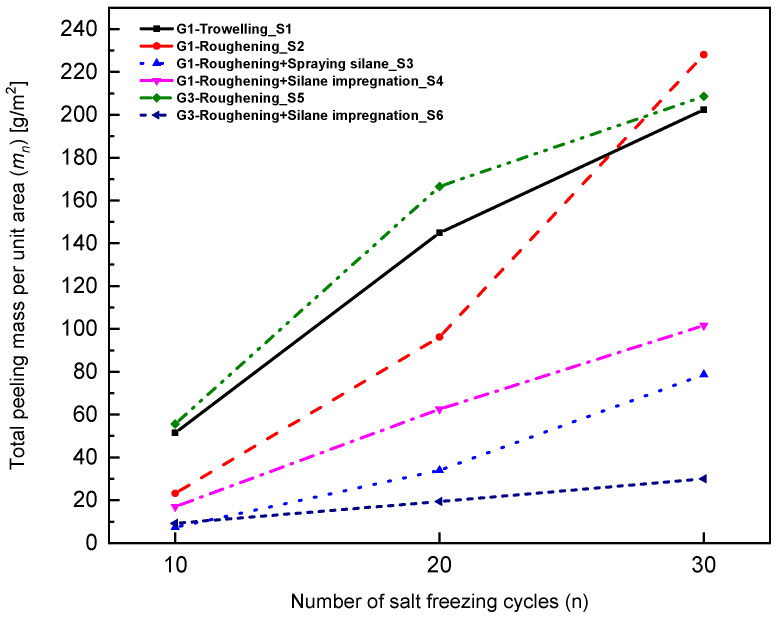
Variation of total spalling mass per unit area with the number of cycles.

**Figure 8:**
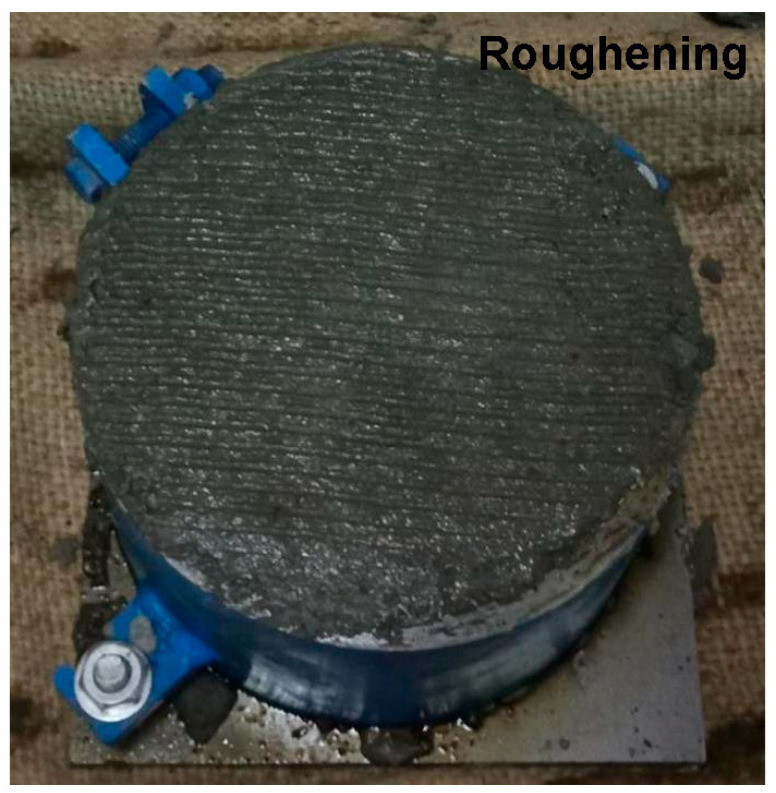
Roughening treatment.

Since G3 had the highest resistance to freeze–thawing cycles, this mix and the control mix G1 were explored further to investigate the effect of surface treatment methods on the salt-freezing performance. Different surface treatment methods were considered and the results are shown in Figure 7. Troweling means that the formed specimen was troweled; roughening means that the surface of the formed specimen was roughened, a basic requirement of the airport pavement (Figure 8); spraying silane means that the forming surface of the concrete specimen was sprayed with 300 g/m^2^ silane; and silane impregnation means that the formed surface of the concrete specimen was soaked in the silane (5 mm) for one day.

According to the requirements of high friction coefficient, the airport pavement needs to be roughened [26]. In addition, to resist the highly cold environment, silane protection is often used in practical projects [29]. In this paper, four surface treatments are carried out for G1 without fiber, namely, troweling, roughening, spraying silane, and silane impression. Two surface treatments are carried out for G3 mixed with FC, roughened only, and roughening + silane impregnated. The results of 30 salt-freezing cycles on the above six groups (S1–S6) are shown in Figure 7.

It is obvious from the experimental results that no matter how the surface is treated and whether fibers are added or not, the total peeling mass of the three groups impregnated or sprayed with silane is less than that of the three groups not sprayed or soaked with silane. This shows that the use of silane can significantly improve the salt-frost resistance of concrete. Compared with S2, the spalling quality of concrete after 30 times of salt freezing was reduced by 65.5% and 55.5%, respectively, in S3 (silane is sprayed) and S4 (silane is soaked). Compared with S5 with S2, the spalling quality of concrete after 30 times of salt freezing was reduced by 8.54%. Compared with S6 with S4, the spalling quality of concrete after 30 times of salt freezing was reduced by 70.5%. It is obvious that fiber can increase the salt-freezing resistance of concrete, and this effect is more significant in specimens with impregnating silane.

### 3.4. Pore Structure Characteristics of Fiber-Reinforced Concrete

**Figure 9:**
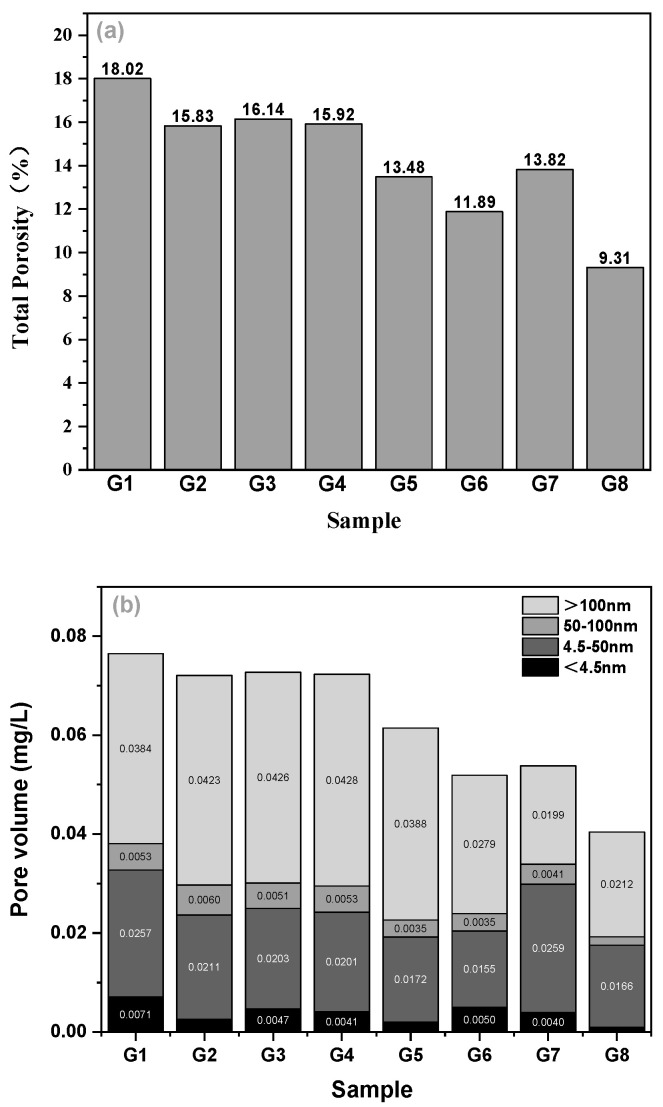
Total porosity (**a**) and absolute pore volume (**b**) of concrete.

**Figure 10:**
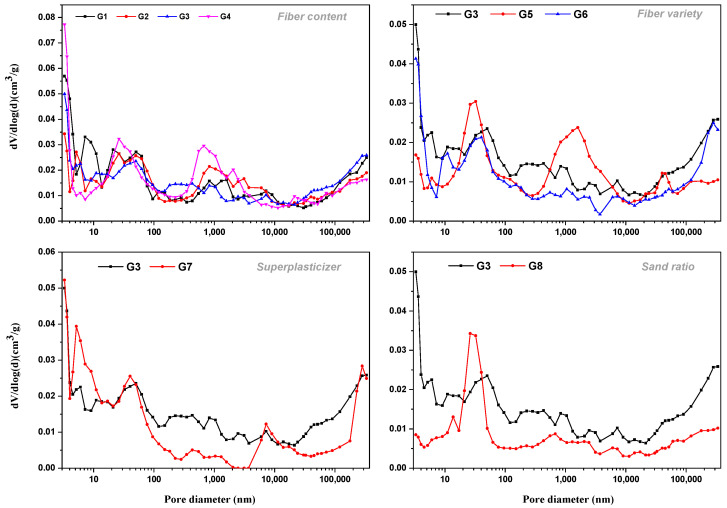
Size distribution of mercury injection voids in concrete.

Figure 9 and Figure 10 show the porosity, pore volume, and pore distribution in all mixes. Cement-based materials are porous materials and the pore characteristics of materials have a decisive impact on their properties. An MIP test was carried out on eight groups of concrete, and important parameters such as total porosity, absolute void volume, and pore distribution can be obtained, as shown in Figure 9a,b and Figure 10 below. The effects of superplasticizer, sand ratio, fiber content, and fiber types on porosity were discussed from different aspects in the following parts: 

#### 3.4.1. Effect of Superplasticizer and Sand Ratio

Compared with G1, the use of air entraining superplasticizer increases the total porosity (G3 and G7), and also increases the pore volume of <4.5 nm, 50–100 nm, and >100 nm except for the pore diameter of 4.5–50 nm. The characteristic pore size—the pore size corresponding to the highest peak value of pore size distribution—of the group without superplasticizer is smaller, which may be more favorable for the concrete to show better mechanical, but not frost, resistance.

From Figure 9, it seems that there is no direct relationship between the sand ratio and the pores of concrete. The sand rate often affects the durability of concrete after hardening by affecting the workability of a concrete mixture. The appropriate sand ratio is that the concrete mixture has good workability and is easy to vibrate and compact. To a certain extent, it can improve the compactness and impermeability of concrete, improve the resistance to the destructive effect of external corrosive media, and effectively reduce the degree of erosion and delay the development speed of erosion. For concrete in a freeze–thaw environment, demonstration of good impermeability depends on the ability of the concrete to resist freeze–thaw damage. If the sand ratio is too large or too small, the fluidity of the concrete mixture will be reduced. On one hand, the concrete is not easy to mix evenly and the homogeneity is poor; on the other hand, during the pouring process, the concrete was not easy to be vibrated and compacted. After pouring, honeycombs and cavities easily formed inside the concrete mix and inside the formwork, and pitted surfaces easily formed on the surface, increasing the porosity inside the hardened concrete. In general, in this experiment, the sand ratio of 0.28 seems to be more favorable than that of 0.3: the porosity of concrete with a sand ratio of 0.28 was lower, and the total porosity was reduced by about 42.3% compared with that of concrete with a sand ratio of 0.3. The absolute pore volume of each size of concrete with a sand ratio of 0.28 was also low, and the characteristic pore size was also less than that of concrete with a sand ratio of 0.3.

#### 3.4.2. Effect of Fiber Contents and Types

When comparing G2, G3, and G4 groups with the G1 group without fiber, it was observed that the addition of fiber into the cement matrix improved the compactness of the matrix. This can be explained by the fact that these micro fibers penetrated into the pores of the pastes and thus reduced the porosity. The porosity of the three groups with fiber was less than that of the G1 group without fiber, and the reduction range was more than 10%. In terms of the fiber types, concrete with basalt fibers showed a lower porosity, which might not be a good option for frost resistance. BF is a typical silicate fiber, which is close to the density of cement concrete. It is easier to disperse and evenly distribute in the concrete during mixing, overcoming the shortcomings of uneven distribution such as agglomeration of other fibers. FC fiber has a large surface area, which restricts the micro-cracks so that they are not connected, but its density is small, the diameter of monofilament is small, and there is a thickening effect, which is not conducive to the vibration compaction of concrete. Therefore, the porosity of FC concrete was the largest among the three fibers, which could be beneficial to resist frost damage.

### 3.5. XRD Results

**Figure 11:**
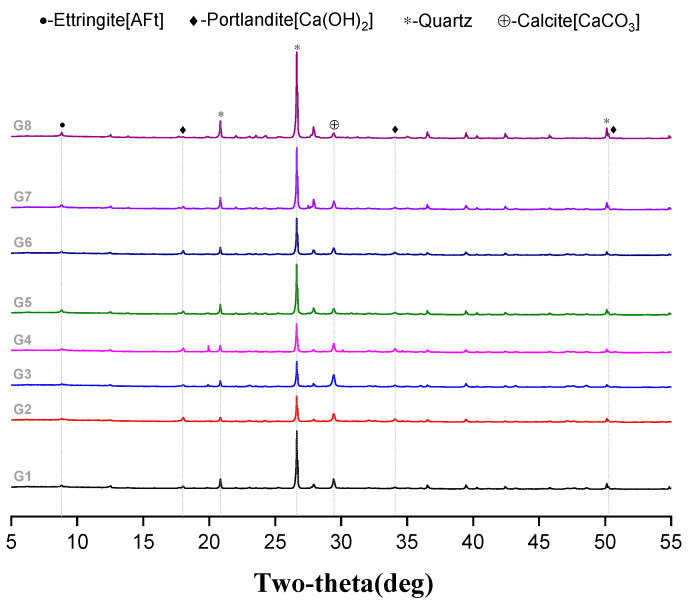
XRD pattern of 8 groups of concrete.

**Figure 12:**
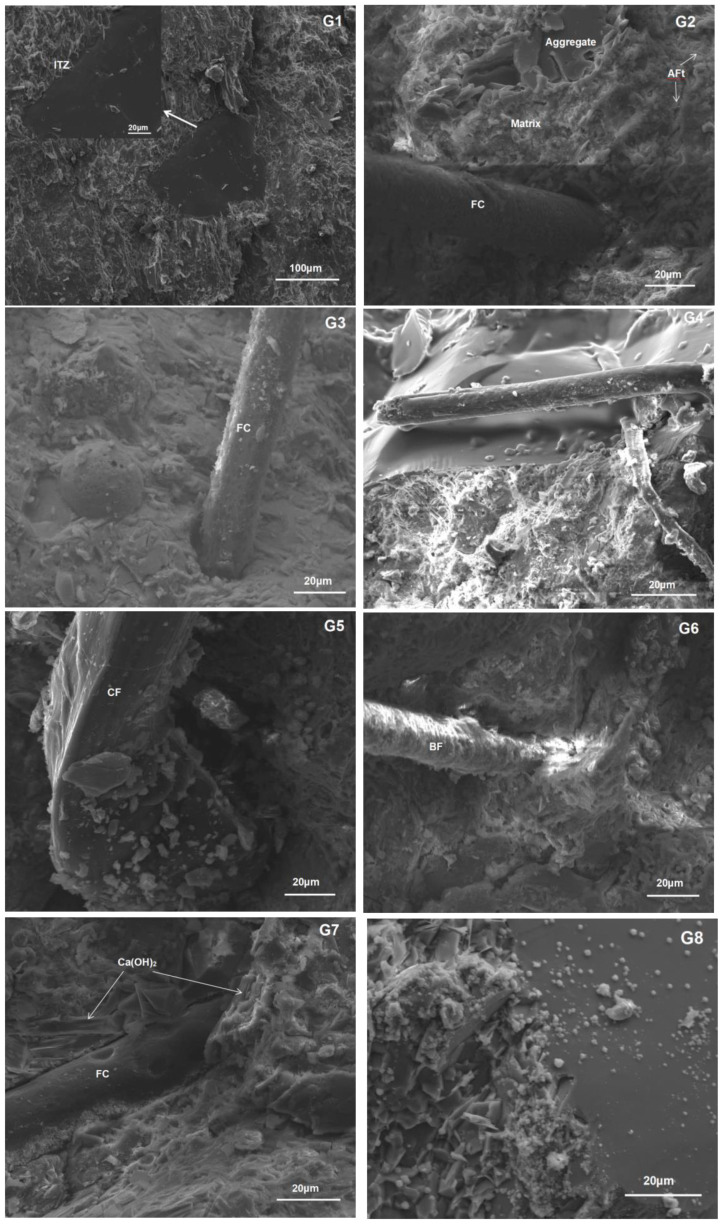
Electron microscope pictures of 8 groups of concrete.

The results of eight groups of XRD for paste sampling in concrete are shown in Figure 11 below. Although the sample was taken to knock out the clean paste as much as possible, it will inevitably be doped with quartz sand. In addition, the main products of cement hydration, calcium hydroxide (portlandite), and AFt can be seen in XRD. This shows that when the type of fiber, the amount of fiber, the use of superplasticizer, and the size of sand ratio changes, the main hydration products in the paste do not obviously change. The hydration products portlandite and AFt did not change greatly and these products can also be seen in the electron microscope results in Figure 12.

In addition, for G8 with a low sand ratio and G7 without a water reducing agent, the XRD results show that the peak value of calcium hydroxide, the hydration product of cement, is lower than that of their respective control groups. It was determined that the use of a low sand ratio without a water reducing agent affects the workability of concrete, and then affects the hydration degree of concrete. The G1 group was not mixed with fiber and the amount of calcium hydroxide was also lower than that of the corresponding control group. It can be considered that the use of fiber in concrete will not affect the hydration of cement, but may promote the hydration degree. According to the consistency of their calcium hydroxide content, it appears that the kind of fiber and the amount of fiber used in G2-G6 fiber-reinforced concrete will not significantly affect the degree of hydration.

### 3.6. Microstructure of Interface Transition Zone

The test results show that after adding fiber, the frost resistance of the concrete is greatly improved. In order to study the modification mechanism of fiber in the concrete matrix, SEM was used to analyze the microstate of the interfacial transition zone (ITZ) of concrete specimens and the interfacial structure characteristics of the fiber-cement paste and the improvement effects were analyzed [30,31,32]. The results are shown in Figure 12. It can be clearly seen that the microscopic morphology of FC (G3), CF (G5), and BF (G6) fibers under the electron microscope is different. The surface of the BF fiber is the roughest, and interacts with the surrounding mortar, which is consistent with its highest flexural strength. By adding an appropriate amount of fiber into the concrete, the fiber is randomly distributed in three dimensions and completely bonded with the concrete. When the concrete cracks appear, the existence of fiber can provide a bridge relay and increase the bonding force inside the concrete, so as to reduce the expansion of large voids and cracks in the process of concrete hardening [33,34]. More importantly, it reduces cracks, changes pore structure, reduces pore connectivity, effectively reduces water transmission channels, makes the damage caused by freezing and thawing relatively independent, and effectively controls the occurrence of freezing and thawing, so as to enhance the frost resistance [4]. In addition, the fibers are overlapped with each other and a fiber frame can be formed. The external bending force is greatly absorbed by the fiber frame in the matrix, and fibers are added to effectively reduce the occurrence of local damage, so as to enhance the flexural strength and frost resistance.

## 4. Discussion

### 4.1. Influence of Fiber Types and Contents on Concrete Strength

The compressive and flexural strength of concrete are the basic mechanical indexes of airport pavement concrete. From the results in Figure 4, it can be seen that the addition of fiber has little effect on the compressive strength, but the flexural strength has been significantly improved. In addition, the reinforcing effect of different fibers on flexural strength is different, showing a positive correlation trend with an elastic modulus of fiber. The fiber with a large elastic modulus has a greater reinforcing effect on the flexural strength of concrete, which may be why the crack resistance effect of high elastic modulus fiber is more obvious [35,36,37,38,39,40,41]. At the same time, the content of fiber has a great influence on the strength of concrete. According to the fiber reinforcement theory, the increase in fiber content up to an optimum content can lead to an increase in the number of fibers per unit volume and the increase in fiber spacing. However, it must be based on concrete compaction [39,42,43,44,45]. If the fiber content is too high (shown as >1.4 kg/m^3^ in this study), it will lead to insufficient mixing and vibration of concrete, resulting in unconsolidated concrete and reduced strength. Therefore, the optimal fiber content recommended in this study is 1.4 kg/m^3^.

### 4.2. Effect of Fiber Types on Frost Resistance of Concrete

Frost resistance is another important technical index of airport pavement concrete. By comparing the test of the relative dynamic elastic modulus and mass loss of concrete mixed with CF and FC fiber and concrete without fiber, it can be concluded that adding fiber can improve the frost resistance of concrete, due to the creation of a mechanical bite between fiber and substrate. The stress suffered in concrete can be borne by the tensile and shear resistance of the fibers, so as to improve the strength of the interface between the fiber and the substrate, which is beneficial to alleviate the freeze–thaw temperature stresses in the concrete and improve the frost resistance [36]. From the perspective of fiber types, the chemical composition of CF fiber belongs to polysaccharide, and the surface is more hydrophobic than the modified FC fiber. Therefore, the thickness of the water film interface is larger, and the microstructure results show that the interface strength is weak (Figure 12 G4). From this point of view, the improvement effect of CF on the frost resistance of concrete is not as good as FC, which agrees with the freeze–thawing results. It can be proved that freeze–thaw cycles impair the fiber/matrix interfacial bond, which is attributed to be the main cause of the degradation of durability [37]. Therefore, the interfacial bonding between fiber and matrix is a performance that should be paid special attention to in airport pavement concrete. The FC fiber used in this experiment realized a high tensile strength (900–1300 MPa), high modulus (8000–13,000 MPa), high toughness, low elongation (10–25%), high alkali resistance (96%), and ultra-large aspect ratio (>300) required by major and special concrete projects, which can fully achieve the bridging function of cement and other mixed materials and effectively prevent concrete cracking [38]. During construction mixing, FC fiber can be evenly distributed in concrete, establish multi-dimensional structure, and be applied in airport runways and other projects, which can improve the durability of airport pavement, realize the excellent performance of freeze–thaw resistance, salt alkali resistance, and corrosion resistance [46,47], and effectively ensure the safety of flight takeoff and landing.

### 4.3. Effect of Surface Treatments on Salt Freezing of Concrete

The results of salt-freezing indicated that silane spraying (S3) is more efficient than silane impregnation (S4). Compared with no silane treatment, the mass loss after 30 salt-freezing cycles in concrete can be reduced by 65.5% by spraying silane. Silane is a small molecular substance, and liquid silane has low viscosity and can penetrate to a certain depth through the open pores on the concrete surface. Under the action of water in the concrete, silane monomer molecules undergo a hydrolysis reaction to produce hydroxysilane [29].

R-Si-(OR’)_3_ + *n*H_2_O→R-Si-(OR’)_3-*n*_(OH)*_n_*+ *n*R’OH (1)

where: *n* ≤ 3 indicates the degree of silane hydrolysis. The hydrolysate hydroxysilane has high activity and the condensation reaction can continue in hydroxysilane. The equation is as follows:

2R-Si-(OH)_3_→R-(OH)_2_SiOSi(OH)_2_-R + H_2_O (2)

The above condensation reaction can occur between hydroxysilane and between hydroxysilane and hydration products on the concrete surface. The former makes silane form a network structure of dimer or even polymer on the concrete surface, and the latter makes silane establish a chemical connection with the concrete surface [48]. Through the two-step reactions (1) and (2), silane forms a stable polymerization network on the surface of concrete pores, to which alkyl R gives hydrophobicity to the surface of concrete pores.

The condensation reaction will cause a dehydration process. A relatively dry concrete (pore) surface is more conducive to reaction (2), and reaction (2) is easier to carry out. 

## 5. Conclusions

In this paper, three types of fiber (FC, CF, and BF) reinforced concrete were prepared. The physical properties, and freeze–thawing and salt-freezing performance were investigated, and the microstructure and pore distribution of different fiber-reinforced concretes were characterized by SEM and MIP. Some favorable measures to improve the frost resistance of pavement concrete are put forward. The main conclusions are as follows:

(1) All fibers improve concrete flexural strength but have little effect on compressive strength. Under the same mass fraction (1.4 kg/m^3^), the flexural strength of FC and BF concrete increased by 4.36% and 4.64% compared with that of ordinary concrete, but CF concrete increased by only 1.17%. The flexural strength of fiber-reinforced concrete is positively correlated with the elastic modulus of fiber.

(2) The use of fiber can improve the frost resistance of concrete, characterized by less mass loss and dynamic modulus loss. After 300 freeze–thaw cycles, the relative dynamic elastic modulus of the control group (G1) was reduced to 0.93, while the relative dynamic elastic modulus of G3 and G5 with fibers was reduced to 0.968 and 0.966, respectively, increasing the relative dynamic elastic modulus by 4.03% and 3.86%, respectively. Compared with CF, FC is more effective in improving concrete frost resistance.

(3) Regardless of fiber types, the total peeling amount of concrete impregnated or sprayed with silane was less than that of concrete not sprayed or impregnated with silane. The use of silane can significantly improve the salt-frost resistance of concrete. After 30 cycles of salt freezing, the spalling amount of concrete sprayed or soaked with silane was decreased by 65.5% and 55.5%, respectively. Furthermore, adding fiber and impregnating silane reduced the spalled concrete by up to 70.5%.

(4) It is recommended to add FC with a content of 1.4 kg/m^3^ in the airport pavement concrete, and apply silane spraying on the exposure surface to protect the concrete. Spraying silane treatment was better than impregnating silane treatment in enhancing the frost resistance of concrete because the dry concrete surface is more conducive to silane condensation reaction in the spraying treatment. Proper surface treatment is more important than the effect of fiber when the concrete is subjected to salt freezing.

## Figures and Tables

**Table 1:** Basic properties of coarse aggregates.

Characteristic	Large Gravel 16–31.5 mm	Small Gravel 4.75–16 mm
Apparent density	2.716	2.731
Water absorption rate	0.63	0.60
Mud content (%)	0.9	0.8
Crushing value (%)	/	17.9

Note: The mud content refers to the content of particles less than 0.075 mm.

**Table 2:** Fiber physical and mechanical properties index.

Fiber Type	Diameter μm	Tensile Strength/MPa	Density g/cm^3^	Modulus of Elasticity/GPa	Elongation %	Length mm
FC	40	1252	1.31	11.7	21	18
CF	18	960	1.10	9	3.5	18
BF	150	1930	2.65	80	3	20

**Table 3:** Experimental mix design.

Group	Cement (kg/m^3^)	Sand (kg/m^3^)	Large Gravel (kg/m^3^)	Small Gravel (kg/m^3^)	Water (kg/m^3^)	Sand Ratio	Fiber Content (kg/m^3^)	Fiber Type	Superplasticizer (kg/m^3^)
G1	330	611.40	784.63	641.97	132.00	0.30	0	0	6.6
G2	330	611.40	784.63	641.97	132.00	0.30	1.0	FC	6.6
G3	330	611.40	784.63	641.97	132.00	0.30	1.4	FC	6.6
G4	330	611.40	784.63	641.97	132.00	0.30	1.8	FC	6.6
G5	330	611.40	784.63	641.97	132.00	0.30	1.4	CF	6.6
G6	330	611.40	784.63	641.97	132.00	0.30	1.4	BF	6.6
G7	330	611.40	784.63	641.97	132.00	0.30	1.4	FC	0
G8	330	570.64	807.05	660.31	132.00	0.28	1.4	FC	6.6

## Data Availability

The data presented in this study are available on request from the corresponding author.
